# Nephroprotective Effect of Black *Panax vietnamensis* var. *fuscidiscus* Against Cisplatin Toxicity

**DOI:** 10.3390/molecules31101586

**Published:** 2026-05-09

**Authors:** Huy-Truong Nguyen, Thi My Duyen Ngo, Mong Kha Tran, Thi Kim Ngan Tran, Truong Tuong Vy, Thi Ngoc Giau Vo, Le Viet Hoang, Danh Duc Ong, Yen Nhi Le Nguyen, Kim Chi Thi Le, Kim Long Vu-Huynh

**Affiliations:** 1 Research Group in Pharmaceutical and Biomedical Sciences, Faculty of Pharmacy, Ton Duc Thang University, Ho Chi Minh City 700000, Vietnam; nguyentruonghuy@tdtu.edu.vn; 2 Faculty of Pharmacy, Ton Duc Thang University, Ho Chi Minh City 700000, Vietnam; ngothimyduyen@tdtu.edu.vn (T.M.D.N.); tranmongkha@tdtu.edu.vn (M.K.T.); kimngantran0503@gmail.com (T.K.N.T.); trtuongvy1012@gmail.com (T.T.V.); vothingocgiau.st@tdtu.edu.vn (T.N.G.V.); hoangleviet.st@tdtu.edu.vn (L.V.H.); ongdanhduc.st@tdtu.edu.vn (D.D.O.); h2100440@student.tdtu.edu.vn (Y.N.L.N.); 3 Department for Facility Management, Ton Duc Thang University, Ho Chi Minh City 700000, Vietnam; lethikimchi@tdtu.edu.vn

**Keywords:** *Panax vietnamensis* var. *fuscidiscus*, cisplatin, nephrotoxicity, ocotillol saponin

## Abstract

*Panax vietnamensis* var. *fuscidiscus*, or PVF, a member of Araliaceae, is a new and high-value variety of Vietnamese Ginseng *(P. vietnamensis* var. *vietnamensis*—VG). PVF shares some similarities in terms of its saponin profile with VG, including protopanaxadiol, protopanaxatriol, and ocotillol saponin. Previous research has revealed that the steaming process significantly increases the bioactivities of VG, especially the renal protective effect. In this study, PVF roots were steamed at a high temperature (120 °C) for 12 h to obtain Black PVF (BPVF). The BPVF extract was tested in both in vitro and in vivo models of cisplatin toxicity to assess its antioxidant and nephroprotective activities. The results showed that the BPVF obtained from the steaming process exhibited the highest antioxidant activity at 12 h. The chemical composition of BPVF is characterized by less-polar saponins such as G-Rg3, -Rg5, and ocotillol genin. The BPVF extract (200 mg/kg) reversed kidney injuries by significantly lowering serum creatinine and blood urea nitrogen (BUN) levels, which had increased due to cisplatin toxicity. The antioxidant effect of BPVF extract also prevented lipid peroxidation by lowering malondialdehyde (MDA) levels and restoring redox balance by increasing glutathione (GSH) content in kidney cells to nearly normal levels, with effects comparable to quercetin. This study provides evidence of BPVF’s therapeutic potential with respect to kidney injuries due to cisplatin toxicity.

## 1. Introduction

Cisplatin is a platinum-based alkylating agent used to treat a wide range of cancers, including head and neck, lung, testicular, ovarian, and cervical cancer. Cisplatin’s anticancer mechanism consists of preventing cancer cells from dividing and growing by damaging their DNA. However, the use of this chemotherapeutic drug is limited due to its toxic effects on the nervous system, liver, bone marrow, gastrointestinal tract, and, in particular, the kidneys. The filtration and excretion of cisplatin in the kidneys result in its accumulation in the renal tubules. Once taken up by renal proximal tubular cells through copper transporter (CTR-1), cisplatin forms reactive species via aquation. This nephrotoxin causes stress and damage in cells by triggering oxidative stress, an inflammatory response, and activating the cell death pathway [1].

Many studies on Korean Ginseng (*Panax ginseng*, KG) have demonstrated the potential nephroprotective effects of processed *P. ginseng* in both in vitro and in vivo models. The processing methods used for White Ginseng—including high-temperature, high-pressure steaming (Sun Ginseng) and fermentation—produce bioactive, less-polar ginsenosides. G-Rk3 and -Rh4, resulting from the hydrolysis of the hydroxy group from protopanaxatriol ginsenosides (PPT), can restore kidney function by reducing serum creatinine and BUN levels and restoring antioxidant defense by increasing renal catalase and superoxide dismutase levels [2,3]. The protopanaxatriol-type ginsenosides (PPD), including G-Rg3, -Rg5, and -Rk1, can significantly reverse cisplatin-induced LLC-PK1 cell damage by inhibiting the expression of inflammatory proteins and regulating apoptosis. The ginsenoside-containing fraction from MG was also found to be able to attenuate elevated serum creatinine levels in cisplatin-treated mice [4]. Fermented black ginseng, the result of repeated heat treatment and fermentation, also ameliorated cisplatin-induced renal damage by inhibiting oxidative stress, inflammation, and apoptosis [5]. Besides PPT and PPD, pseudo-ginsenoside RT5, an ocotillol-type (OT) saponin isolated from *P. quinquefolius*, can also ameliorate cisplatin-induced kidney damage by inhibiting oxidative stress and reducing tubular apoptosis without interfering with the anti-tumor efficiency of the chemotherapy reagent [6].

Discovered in 1973, Vietnamese Ginseng (*P. vietnamensis* var. *vietnamensis*—VG) is the most recently identified *Panax* species to be found on Ngoc Linh Mountain in central Vietnam. VG is characterized by the surprisingly high content of saponin in PPT (e.g., ginsenoside Rg1, Re, Rh1, and notoginsenoside R1), PPD (e.g., G-Rb1, Rc, and Rd), and the OT-skeleton, containing over 5% of majonoside R2 (M-R2) [7,8,9]. In 2003, Zhu et al. [10] found a new variety of *P. vietnamensis* in Yunnan, South China, and named it Ye-Sanchi, with the scientific name *P. vietnamensis* var. *fuscidiscus* (PVF). In 2014, Phan Ke Long et al. [11] reported the distribution of PVF in Lai Chau Province, Vietnam, and used Lai Chau Ginseng as an alternative name for Ye-Sanchi. Similar to VG, PVF is a herbaceous plant with an erect aerial stem, palmately compound leaves, and red berries with a single black spot at their apex. The PVF usage part consists of scarred rhizomes and developed, branched roots (Figure S1). Nguyen Truong Huy et al. [12] determined that PVF shared saponin profiles similar to those of VG, including high levels of G-Rg1, -Rb1, M-R2, etc. However, the high content of vina-ginsenoside R2 (V-R2) in PVF serves as a marker with which to distinguish this variety from VG.

Our recent study demonstrated that steam processing at high temperatures and pressures could significantly enhance the nephroprotective effect of VG. From processed VG extract, six rare PPD-type ginsenosides, including 20 (*S, R*)-G-Rg3, 20 (*S, R*)-G-Rh2, -Rk1, -Rg5, and ocotillol genin, were isolated through nephroprotective-effect-guided extraction and found to exhibit a kidney-protective effect against cisplatin toxicity in an in vitro system [13]. Moreover, panaxynol, a polyacetylene isolated from VG, also exhibited a protective effect against cisplatin-induced damage in both in vitro and in vivo models, reducing apoptosis and inhibiting the inflammatory response [14]. Due to the similarity in the ginsenoside profiles of VG and PVF, along with the extremely high content of the OT-type saponin (the precursor of ocotillol genin), it is hypothesized that high temperature and pressure steaming could increase the nephroprotective effect of this variety of VG.

In this study, we aimed to investigate the effect of the steaming process on the chemical composition and biological activity of PVF. Specifically, we developed Black *P. vietnamensis* var. *fuscidiscus* (BPVF) by steaming PVF roots at high temperatures and analyzed the changes in saponin profiles and antioxidant capacity. Furthermore, the potential nephroprotective effect of the optimized BPVF extract was evaluated against cisplatin-induced toxicity using both in vitro and in vivo models.

## 2. Results

### 2.1. Saponin Composition of BPVF Analyzed via UPLC-QToF-MS

**Figure 1:**
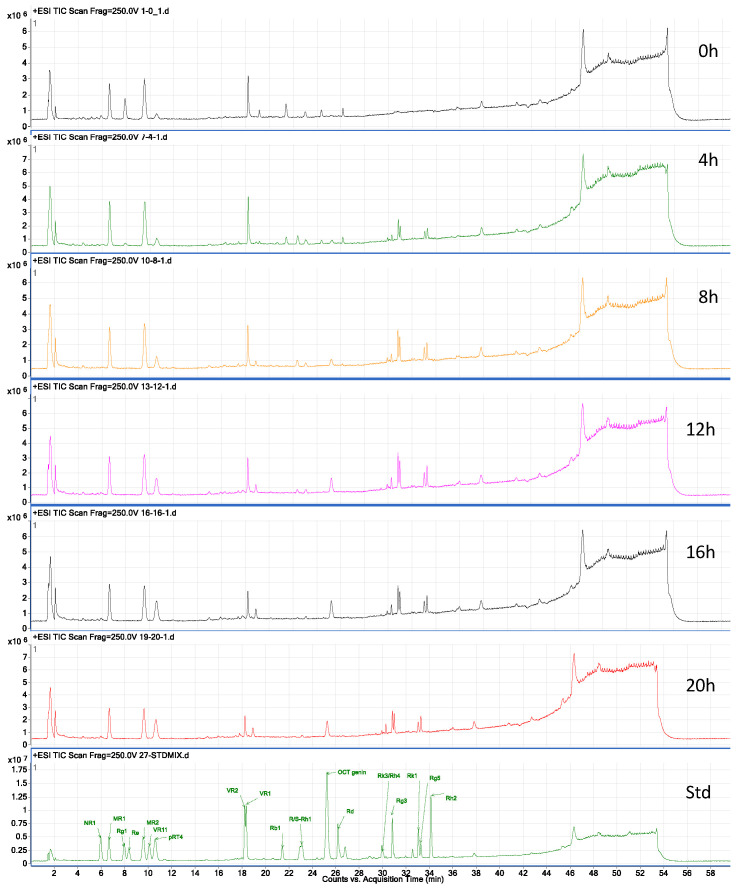
Total ion chromatogram of BPVF extracts analyzed via UPLC-QTOF-MS.

**Figure 2:**
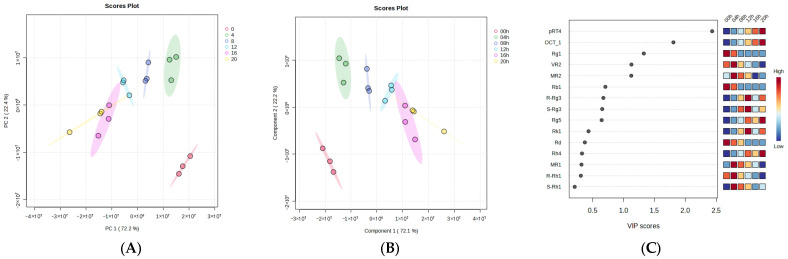
PCA (**A**) and PLS-DA (**B**) of BPVF steaming at 0–20 h, VIP score of top 15 saponins (**C**).

In the chromatogram of the different steaming time points (Figure 1), a contradictory trend in peak intensity is clearly evident. For instance, the first half of the chromatogram, from retention time 4 to 18, shows a gradual decrease in peak intensity from 0 to 20 h of steaming time. In contrast, the remaining half of the chromatogram, from retention time 18 to 38, clearly shows a gradual increase in peak intensity from 0 to 20 h of steaming time. This clearly reflects not only changes in concentration but also possible chemical transformations of the compounds during heating. To better understand these changes, we subjected the resulting data to Principal Component Analysis (PCA), which revealed that Principal Components (PC) 1 and PC2 accounted for a total explained variance of 94.6%, with PC1 alone accounting for 72.2%. The results clearly showed that the 00 h, 04 h, and 08 h groups are well separated, whereas the 12 h, 16 h, and 20 h groups somewhat overlap (Figure 2A), indicating that the chemical changes among the 00 h, 04 h, and 08 h groups are significantly greater and thus well separated. In contrast, the chemical changes in the 12 h, 16 h, and 20 h groups are less significant and hence clustered together. This result suggests that the saponin composition did not change when the steaming time was increased from 12 to 20 h. Next, to pinpoint the markers that corresponded to these chemical changes during the heating process, Partial Least Squares-Discriminant Analysis (PLS-DA) analysis was introduced to maximize the differences (Figure 2B) and derive the markers.

Consequently, we extracted 55 markers with a Variable Importance in Projection (VIP) score > 1, including ocotillol-type saponin, pseudo-ginsenoside RT4 (p-RT4, VIP = 2.43), ocotillol genin (OT, VIP = 1.81), M-R2 (VIP = 1.13), and the PPT-type ginsenoside G-Rg1 (VIP = 1.33). Among these markers, p-RT4 and OT were highly abundant in BPVF at 12–20 h of steaming and may contribute to this extract’s kidney-protective effect. The PPD-type ginsenoside had a VIP score ranging from 0.5 to 1.0, including G-Rb1 (VIP = 0.71), 20(*R*)-G-Rg3 (VIP = 0.67), 20(*S*)-G-Rg3 (VIP = 0.66), and G-Rg5 (VIP = 0.65), which were most prevalent in BPVF after 12 h of heating (Figure 2C).

### 2.2. Antioxidative Effects of BPVF After Different Steaming Durations

**Figure 3:**
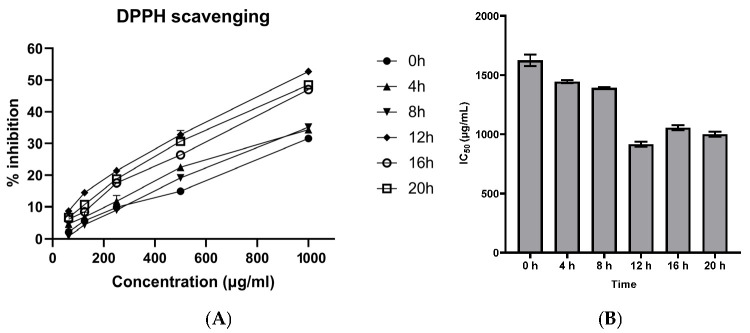
DPPH free-radical-scavenging activity (**A**) and IC_50_ value (**B**) of PVF at different heating times. Results are expressed as means ± SDs (*n* = 3).

The antioxidant activity of BPVF obtained from steamed PVF was evaluated using the DPPH (2,2-diphenyl-1-picrylhydrazyl) method. The results in Figure 3A show that the DPPH free-radical scavenging activity of the BPVF extract increased with heating time. When the steaming time increased to 0 h and 12 h at the highest concentration, namely, 1000 µg/mL, the inhibition rates increased to 31.5% and 52.7%, respectively. There were no significant differences in the antioxidant activity of the extract after 8 and 20 h of steaming. The IC_50_ of BPVF at 12 h was 914.9 ± 22.59 µg/mL, which is 1.72 times lower than that of the raw PVF extract (1622.62 ± 46.66 µg/mL). However, when the steaming duration was increased to 20 h, the IC_50_ value increased slightly to 999.40 ± 22.0 µg/mL, but the change was not statistically significant. From the results, it can be concluded that 12 h of steaming yielded the BPVF extract with the highest antioxidant activity, which was evaluated for its kidney-protective effect against cisplatin toxicity.

### 2.3. In Vitro Nephroprotective Effect of BPVF

**Figure 4:**
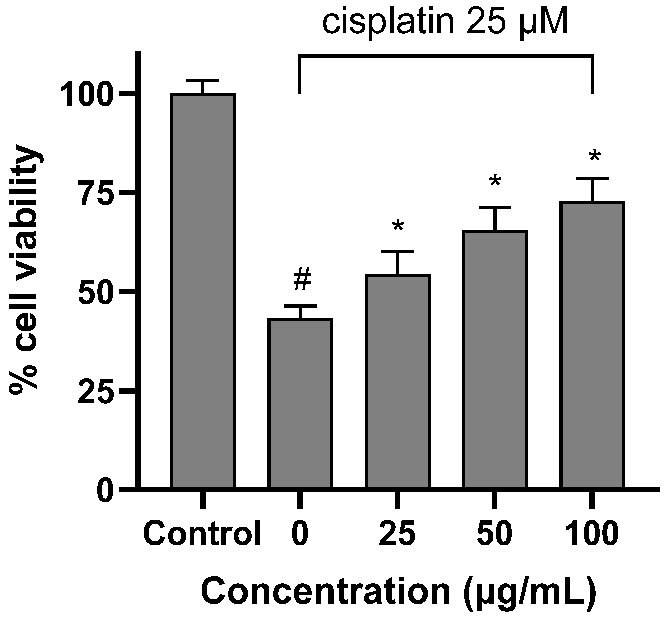
BPVF extract, after 12 h of steaming, recovered LLC-PK1 cells lost due to cisplatin toxicity in dose-dependant manner. Data are expressed as mean ± SD (*n* = 3). One-way ANOVA showed a significant effect (*p* < 0.001). # *p* < 0.05 vs. control (without cisplatin); * *p* < 0.05 vs. previous concentrations of the test sample co-treated with 25 µM cisplatin.

The BPVF extract with the highest antioxidant activity was selected for an evaluation of its nephroprotective effect against cisplatin toxicity. To evaluate the kidney recovery capacity of the BPVF extract steamed for 12 h, LLC-PK1 cells were treated with 25 µM cisplatin in the presence of 25, 50, and 100 µg/mL of extract, and cell viability was detected using water-soluble tetrazolium salt WST-8 reagent. As shown in Figure 4, after 24 h of incubation, cisplatin (25 µM) significantly decreased cell confluence to 43.3 ± 3.04% (*p* = 0.003). When the cells were co-treated with the PVF extract at doses of 50 and 100 µg/mL, cell viability increased to 65.5 ± 5.53% (*p* = 0.025) and 72.7 ± 5.76% (*p* = 0.020), respectively. These results suggest that steamed PVF extract could protect the kidneys against cisplatin-induced damage via an antioxidant mechanism.

### 2.4. In Vivo Kidney-Protective Effect of BPVF

**Figure 5:**
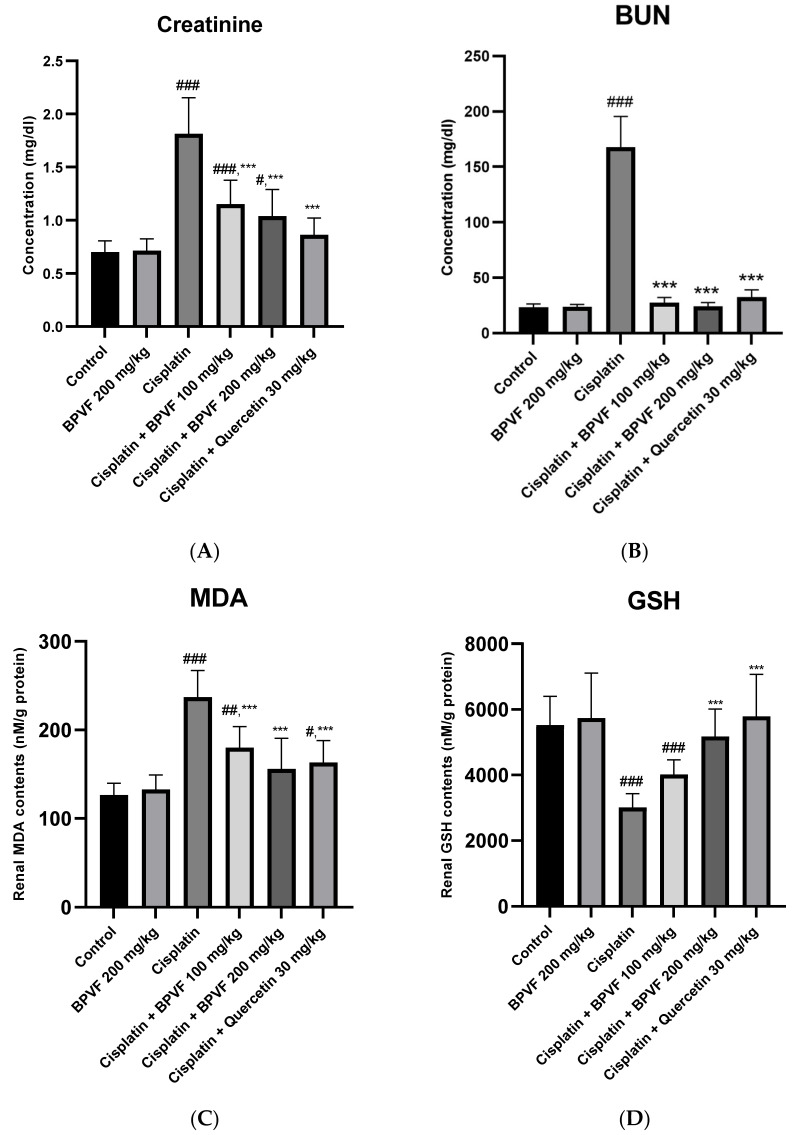
Effects of BPVF on renal function and oxidative stress markers in cisplatin-treated mice. (**A**) Plasma creatinine, (**B**) blood urea nitrogen (BUN), (**C**) renal malondialdehyde (MDA), and (**D**) glutathione (GSH) levels. Data are expressed as mean ± SD (*n* = 8). One-way ANOVA indicated significant differences among groups (A: *p* < 0.001; B: *p* < 0.001; C: *p* < 0.001; D: *p* < 0.001). # *p* < 0.05, ## *p* < 0.01, and ### *p* < 0.001: compared to a normal control; *** *p* < 0.001: compared to the cisplatin-only group. Cisplatin significantly increased creatinine, BUN, and MDA levels and reduced GSH levels compared with the normal control group (*p* < 0.001). Treatment with BPVF (100 and 200 mg/kg) significantly ameliorated these alterations compared to the cisplatin group (*p* < 0.05–0.001). Notably, BPVF at 200 mg/kg showed comparable effects to quercetin in reducing creatinine (*p* = 0.113 vs. quercetin) and restoring oxidative stress markers. BPVF administered alone did not significantly alter any parameter compared to the normal control (*p* > 0.05).

The nephroprotective effect of BPVF was evaluated using a cisplatin-induced acute kidney injury model by assessing renal function biomarkers and oxidative stress indicators. As shown in Figure 5, BPVF alone (200 mg/kg) did not induce significant changes in creatinine, blood urea nitrogen (BUN), malondialdehyde (MDA), or glutathione (GSH) levels compared with the normal control group (*p* > 0.05), indicating the absence of intrinsic toxicity. Clinically, cisplatin nephrotoxicity is often observed after 10 days of cisplatin administration. It is characterized by a lower glomerular filtration rate and higher serum creatinine levels. As shown in Figure 5A,B, plasma creatinine and BUN levels in the cisplatin-treated control mice were significantly elevated (159.0% and 618.2%, respectively) compared to the control group (*p* < 0.001), reflecting renal damage induced by cisplatin. Quercetin (30 mg/kg) significantly reduced plasma creatinine and BUN levels compared with those in the cisplatin-only group (*p* < 0.001) and returned to normal values compared with the normal control (*p* = 0.302 and *p* = 0.553, respectively). The administration of BPVF (100 mg/kg and 200 mg/kg) to the cisplatin-treated mice led to a statistically significant decrease in creatinine content compared with the cisplatin-only control. In the former group, BPVF (200 mg/kg) decreased plasma creatinine levels, which were similar to those of quercetin (30 mg/kg) (*p =* 0.113). However, BPVF at both doses (100 mg/kg and 200 mg/kg) failed to restore plasma creatinine levels to normal levels relative to the physiological controls in the cisplatin-induced kidney injury model (*p* = 0.001; *p* = 0.015, respectively).

Figure 5C,D show oxidative damage in the kidneys induced by cisplatin, as evidenced by a significant increase in MDA (86.9%) and a reduction in GSH (45.6%) content in renal tissues relative to the normal control (*p* < 0.001). Administration of BPVF (100 mg/kg and 200 mg/kg) in cisplatin-subjected mice led to a statistically significant decrease in MDA content and an increase in GSH content relative to the cisplatin-only control (*p* < 0.001). In particular, BPVF (200 mg/kg) restored MDA and GSH levels to nearly normal values compared with the control group that did not undergo cisplatin treatment.

## 3. Discussion

*P. vietnamensis* var. *fuscidiscus* is well-known for its high content of PPD, PPT, and, especially, OT-type saponin (M-R2 and V-R2). Steaming at 120 °C for 12 h cleaves the sugar moiety at C-20 of the original PPD- and PPT-type polar ginsenosides, completely converting them into less-polar forms. Similarly, V-R2 possesses a carbonyl group at the C-6 position of the sugar chain; this group can readily be eliminated to form M-R2. However, the glycosidic linkage between the two sugar moieties of M-R2 is much more resistant to heat hydrolysis. Therefore, M-R2 is partly converted into p-RT4, which is then converted into OT genin. Interestingly, the presence of the glycoside-form OT-type saponin (V-R2, M-R2, and p-RT4) and OT genin, along with less-polar ginsenosides, distinguishes BPVF from other processed *Panax* species, such as Red Ginseng. The differences in chemical composition may play an essential role in BPVF’s bioactivity.

The DPPH assay was employed as a rapid, widely used method for preliminary screening of radical-scavenging activity, allowing comparisons among samples processed under different conditions. Using DPPH screening assays, the antioxidant activity of BPVF increased with steaming time, reaching a maximum at 12 h. This result closely aligns with the findings of Van et al. (2014) [15], who reported that PV’s DPPH scavenging activity increases gradually with an increase in steaming time and reaches a plateau after 12–14 h of steaming. The antioxidant effect of BPVF may be the combination of complex compositions in the extract. Firstly, similarly to processed PV, the antioxidant activities of BPVF may stem from less-polar PPD-type saponins, such as G-Rg3. Xiaojie Wei et al. (2012) [16] validated the antioxidant activity of 20(*S*) and 20(*R*)-G-Rg3 by inhibiting cyclophosphamide-induced oxidative stress in mice. Moreover, the high content of ocotillol-type saponins in the BPVF extract, including V-R2, M-R2, p-RT4, and ocotillol genin, may also contribute to its antioxidant activity. In a review by Juan Liu et al. (2017) [17], pseudoginsenoside F11 was found to inhibit free-radical formation and stimulate the release of endogenous antioxidants in Parkinson’s disease. p-RT5, another ocotillol-type saponin, also reduces renal tubular damage caused by cisplatin by increasing antioxidant levels. However, further research is needed to confirm the antioxidant activity of ocotillol genin, the product of the hydrolysis of ocotillol saponins, such as M-R2. In addition, the high-temperature, prolonged steaming process results in a darker, blacker color of the BPVF root compared to the raw one. This color change is due to the presence of Maillard-reaction products. The combination of amino acids and glycosides in Red Ginseng has been proven to exert strong free-radical-scavenging activities through in vitro experiments [18]. Jun-Jie Zhang et al. (2023) [19] also found high antioxidant efficiencies of arginyl-fructosyl-glucose in Red Ginseng against mitochondrial dysfunction resulting from ROS in a mouse brain-aging model. However, using a single antioxidant assay is a limitation, as additional methods such as ABTS or FRAP could provide a more comprehensive evaluation of antioxidant capacity.

Cisplatin was approved by the FDA in 1978 for the treatment of solid tumors such as testicular, ovarian, and bladder cancer. However, nephrotoxicity is one of the primary side effects that limit its use. In this study, the nephroprotective effect of BPVF extract was confirmed in vitro and in vivo. In our in vitro cell viability experiment, cisplatin caused significant cell death in the LLC-PK1 cell line. Co-treatment with BPVF extract significantly reversed cell damage in a concentration-dependent manner. As mentioned, the nephrotoxicity of cisplatin is a product of oxidative stress and the inflammatory response. The antioxidative activity of BPVF, as demonstrated via the DPPH scavenging assay, may help mitigate the free radicals generated by cisplatin. As a result, kidney cells may negate the oxidative damage, as evidenced by the increased cell viability (Figure 4). As shown in Figure 1, BPVF had a high content of less-polar saponins in the PPD skeleton, including G-Rg3, Rk1, and Rg5. Our previous study discovered that these compounds exhibited a strong kidney-protective effect against cisplatin toxicity [13]. Moreover, as a heavy metal-containing reagent, cisplatin-induced nephrotoxicity is closely associated with inflammatory activation and immune dysregulation, which contribute to renal tubular injury [20]. The presence of high levels of ocotillol-type saponins such as M-R2, V-R2, p-RT4, and ocotillol genin may play a key role in reducing the inflammatory response triggered by cisplatin. This hypothesis is strongly supported by the study by Jeong et al., which indicated that M-R2, p-RT4, and ocotillol genin can suppress the expression of the inflammatory cytokines TNF-α and IL-1β in macrophages stimulated by lipopolysaccharide. Notably, the ocotillol genin is not found in raw PVF, and it is mainly produced by human gut microbiota. However, the diversity of intestinal flora among individuals may affect the production of ocotillol genin. The presence of ocotillol genin in BPVF extract at a significant level reduces the gut microbiota’s dependence on the output of less-polar ocotillol-type saponins. This advantage could result in a more stable anti-inflammatory and nephroprotective effect.

An in vivo study was conducted in an animal model to confirm the in vitro nephroprotective effect of BPVF. At the clinical level, an acute kidney injury caused by cisplatin administration is characterized by a reduction in the glomerular filtration rate, leading to higher BUN and serum creatinine levels [21]. BPVF extract significantly lowers both kidney indices. In particular, the test sample could return BUN values to nearly normal levels, as with quercetin, which has been reported to be able to protect the kidney from cisplatin toxicity [22]. Therefore, co-treatment with BPVF could significantly restore kidney function reduced by cisplatin-induced nephrotoxicity. One mechanism of cisplatin nephrotoxicity is oxidative stress. Cisplatin depletes the antioxidant system by decreasing levels of antioxidant enzymes [superoxide dismutase (SOD), catalase, glutathione reductase]. The intracellular metabolism of cisplatin into GSH-conjugate nephrotoxin also results in GSH depletion and alters the redox state in kidney cells. Cisplatin-induced oxidative stress also causes lipid peroxidation and increases MDA levels [23]. Our results show that BPVF extract significantly increased GSH levels in kidney tissue, with the resulting levels being similar to those in the control group. This result suggests that BPVF may help restore the redox balance in kidney tissue. The reduction in MDA levels in the group subjected to co-treatment with BPVF and cisplatin also demonstrated that the extract can prevent the lipid peroxidation induced by cisplatin. These findings suggest that BPVF’s antioxidant effect plays a crucial role in its nephroprotective properties. Beyond oxidative stress, kidney injury is closely linked to systemic metabolic disturbances, including alterations in protein, lipid, and carbohydrate metabolism, as evidenced by recent spectroscopic profiling studies [24]. Similar to other *Panax* species, PVF is known as an adaptogen that can help maintain optimal body homeostasis. This suggests that BPVF’s ability to restore redox balance may also reflect its potential to modulate underlying metabolic pathways associated with renal dysfunction.

Many studies have confirmed that steamed ginseng has strong anti-cancer effects, which are mainly a product of the less-polar ginsenosides produced by heat [25]. Additionally, Van et al. (2014) [15] also reported that the anti-proliferative effect of VG increased with an increase in steaming time. This suggests that BPVF, with its highly similar saponin profile, may be a potential anti-cancer reagent. Thus, co-treatment with BPVF and cisplatin may enhance the anti-tumor effect of the platinum-based reagent while also mimicking BPVF’s nephroprotective effect, thereby reducing the drug’s side effects on the kidneys.

Although this study demonstrates biochemical recovery of kidney function through serum indices (creatinine and BUN) and oxidative stress markers (MDA and GSH), it also has limitations. First, due to constraints on sample availability, histopathological examination (e.g., H&E staining) intended to visually confirm structural protection was not performed. Second, specific molecular mechanisms regarding cell death pathways (such as apoptosis or necrosis) were not verified via Western blotting or TUNEL staining. We recommend addressing these aspects in future studies to foster a comprehensive understanding of BPVF’s nephroprotective mechanism.

## 4. Materials and Methods

### 4.1. Materials

Fresh PVF roots were supplied by Lai Chau Ginseng Cooperative (Lai Chau, Vietnam). The fresh roots (500 g) were dried in a drying oven at 40–60 °C for 3 days to obtain dry roots. A portion of the dry roots (10 g) was ground to obtain a powder with a grain size of less than 1 mm for the in vitro experiment. The leftover whole roots (80 g) were used for the in vivo test.

### 4.2. Reagents and Apparatus

LLC-PK1 cells were supplied by ATCC, Manassas, VA, USA. Cisplatin, quercetin, Ellman’s reagent, and DPPH were purchased from Sigma-Aldrich (St. Louis, MO, USA). WST-8 reagent (Ez-Cytox) was supplied by DoGenBio (Seoul, Korea). The Creatinine Assay Kit was purchased from Human (Wiesbaden, Germany), and the BUN Assay Kit was obtained from MyBioSource (San Diego, CA, USA). Reagents for the cell cultures, including DMEM Glutamax I, fetal bovine serum, and antibiotics, were purchased from Gibco (Grand Island, NY, USA). All other chemicals used were of analytical grade. The colorimetric assay was conducted using a SpectraMax 190 microplate reader (Molecular Devices, San Jose, CA, USA).

### 4.3. Methods

#### 4.3.1. Steaming VG Powder at High Temperatures for Different Durations

Steaming was performed as previously described, with minor modifications [13]. PVF powder (100 mg) was placed into a 20 mL stainless-steel vessel, and 1 mL of distilled water was added. The vessels were tightened and heated at 120 °C for 2–20 h. After being steamed, the mixtures were dried under a nitrogen stream and extracted 6 times with 3 mL of 100% methanol. The extract was evaporated under vacuum and then dissolved in DMSO to obtain a stock solution of BPVF at 100 mg/mL for further in vitro research.

#### 4.3.2. Analysis of BPVF Using UPLC-QTOF-MS

The BPVF extracts were analyzed via UPLC-QTOF-MS following the protocol established in our previous study [12]. This analysis was performed using the Agilent Infinity 1290 system coupled with a 6456 QTOF detector (Agilent Technologies, Santa Clara, CA, USA) in negative mode, with a scan range of *m/z* 100–1700 Da, a fragmentor voltage of 350 V, a capillary voltage of 3500 V, a nebulizer gas speed of 5 L/min, and a gas temperature of 300 °C. The BPVF extract was prepared at a concentration of 1 mg/mL and separated on a Phenomenex Kinetex C18 column (50 × 4.6 mm, 2.7 µm) using a mobile phase of acetonitrile (A) and 0.1% aqueous formic acid (B). The gradient elution program was as follows: 0–10 min—20.5% (A); 10–15 min—20.5–30% (A); 15–23 min—30% A; 23–30 min—30–40% (A); and 30–40 min—40–95% (A). The column was washed with 95% (*v/v*) acetonitrile for 5 min and then equilibrated with the initial mobile phase for 5 min. A randomized injection order was applied for data acquisition. For system equilibration, six QC samples were injected at the start of the run. Thereafter, two QC samples were injected for every eight analytical samples, one before and one after, except in the final segments, wherein only four samples were placed between each QC pair.

#### 4.3.3. Multivariate Statistical Analysis

The UPLC-QTOF-MS data were initially examined using Principal Component Analysis (PCA) to visualize the underlying structure and identify potential outliers. Afterward, Partial Least Squares Discriminant Analysis (PLS-DA) was applied to investigate group differences and identify markers (VIP > 1). This multivariate statistical analysis treats all chromatograms as a whole and simultaneously evaluates all metabolic characteristics, thereby enabling the identification of interactions and relationships among them. As a result, this multivariate statistic may provide a more comprehensive overview of this dataset than a univariate statistic. All the analyses were carried out using MetaboAnalyst 5.0 [26].

#### 4.3.4. Evaluation of the Extracts’ Antioxidative Effects

The antioxidant effects of BPVF extracts and fractions were evaluated using the DPPH (2,2-diphenyl-1-picrylhydrazyl) method. DPPH was dissolved in methanol to obtain a 0.2 mM solution. A stock solution of extracts at 100 mg/mL was prepared in DMSO and diluted in methanol to obtain a series of concentrations ranging from 200 to 1000 µg/mL. Quercetin was used as a positive control at concentrations ranging from 1.25 to 15 µg/mL. Methanol was used as a blank. The DMSO concentration was kept at 1% in all the test samples and the blank. An aliquot of 100 µL of the samples or the blank was placed into a 96-well plate, and 100 µL of DPPH solution was added to each well. The reaction mixture was left in the dark at room temperature for 30 min before being measured in a microplate reader at 517 nm. The percentage of DPPH free-radical-scavenging was calculated using Equation (1).

(1) $$\%\mathrm{DPPH}\;\mathrm{radical}\;-\;\mathrm{scavenging}\;\mathrm{activity}=\frac{A_{0}-A_{1}}{A_{0}}\;\text{×}\;100$$

where A_0_ is the absorbance of the blank, and A_1_ is the absorbance of the samples. The percentage of inhibition was plotted against the concentration of the extracts or the positive control. The IC_50_ was calculated based on the graph. The experiment was triplicated at each concentration.

#### 4.3.5. In Vitro Nephroprotective Assay of BPVF

LLC-PK1 cells were cultured in DMEM-Glutamax-I medium containing 10% fetal bovine serum and 1% antibiotics (100 units/mL of penicillin G and 100 µg/mL of streptomycin). The cells were kept in a 5% CO_2_ humidified incubator at 37 °C. After reaching 80% confluence, the cells were seeded into a 96-well plate at a concentration of 1 × 10^4^ cells per well. The cells were incubated for 24 h to promote adhesion, and the medium was then removed. The cells were then treated with BPVF extract at a concentration of 25–200 µg/mL. After 2 h of incubation, 10 µL of 250 µM cisplatin was added to each well, and the plates were incubated for an additional 24 h. After incubation, 10 μL of WST-8 was added to each well. After 3 h of incubation, cell viability was assessed by measuring absorbance at 450 nm using a microplate reader. The experiment was conducted in triplicate to ensure the results were reproducible.

#### 4.3.6. In Vivo Kidney-Protective Effect of BPVF

Whole PVF roots (80 g) were steamed at 120 °C for 12 h in an autoclave. The 12 h steaming condition was selected for in vivo evaluation based on preliminary antioxidant screening, which demonstrated maximal radical-scavenging activity and optimal IC_50_ values compared to other steaming durations. The steamed roots were dried in an oven at 40–60 °C. The resulting dry roots were ground and sieved to obtain a powder with a particle size less than 1 mm. The powder was then extracted using a Soxhlet apparatus with 96% ethanol for 48 h. The extract was dried under a vacuum to obtain the dry BPVF extract.

The animal experiment was carried out with the approval of the Animal Ethics Committee of Ton Duc Thang University (TDTU-AEC) under number 12/HDTVDD. We used 6-week-old male *Swiss albino* mice weighing 25 ± 2 g in the experiment to evaluate cisplatin nephrotoxicity. The mice were housed under stable conditions at 28 ± 2 °C and 65 ± 5% humidity, with a 12 h light/dark cycle. The animals had free access to water and food and were allowed to acclimatize for at least 5 days before the experiment. The oral or injection volume was 10 mL/kg of body weight. The mice were randomly divided into 6 groups (n = 8) and assigned to the following treatment groups: group 1, vehicle-treated mice given water; group 2, non-cisplatin-treated mice administered BPVF extract (200 mg/kg) in an aqueous solution orally for 8 days; group 3, cisplatin-treated mice given water alone; group 4, cisplatin-treated mice administered BPVF extract (100 mg/kg) in an aqueous solution orally for 8 days; group 5, cisplatin-treated mice administered BPVF extract (200 mg/kg) in an aqueous solution orally for 8 days; and group 6, cisplatin-treated mice administered quercetin (30 mg/kg) in an aqueous solution orally for 8 days [4,27]. Acute kidney injury was induced in groups 4–9 by a single intraperitoneal injection of cisplatin in saline (0.9%) in a single dose of 15 mg/kg on day 5 [14]. On day 8, blood samples were collected from the tail vein. Whole blood was allowed to clot by leaving it undisturbed at room temperature. The clot was removed via centrifuging at 2000× *g* for 10 min in a refrigerated centrifuge. The resulting supernatant was used to determine creatinine and blood urea nitrogen (BUN) levels using commercial kits. Kidneys were harvested and homogenized in a 1.15% KCl buffer at 13,000 rpm. The tissue homogenate was used to determine renal malondialdehyde (MDA) and glutathione (GSH) levels using previously reported protocols [28,29]. The MDA and GSH levels were calculated from the standard curve of y = 0.0796x + 0.0035 (R^2^ = 0.9992) and y = 0.0043x − 0.0033 (R^2^ = 0.9997), respectively, expressed as nM/g protein.

#### 4.3.7. Data Analysis

The data were expressed as means ± SDs (Standard Deviations), and statistical analysis was performed using a One-way ANOVA followed by the Student–Newman–Keuls test (SigmaStat 3.5). Test results were considered statistically significant at the 95% confidence level (*p* < 0.05) compared with the respective normal control, cisplatin-only control, or positive control.

## 5. Conclusions

The results of this study demonstrate that Black *Panax vietnamensis* var. *fuscidiscus* obtained via steaming at high temperatures exerts a significant nephroprotective effect against cisplatin-induced kidney injury through antioxidant mechanisms. BPVF reduced oxidative stress by restoring the redox balance and improved functional biomarkers in both in vitro and in vivo models. The unique enrichment of less-polar ocotillol-type saponins distinguishes BPVF from other processed *Panax* products. These findings suggest that BPVF is a promising functional material for improving chemotherapy-induced nephrotoxicity. We recommend conducting further investigations to elucidate the specific molecular mechanisms underlying the nephroprotective effects of BPVF, including the regulation of the MAPK and NF-κB signaling pathways.

## Data Availability

The data presented in this study are available in the main article and its Supplementary Materials.

## Supplementary Materials

The following supporting information can be downloaded at https://www.mdpi.com/article/10.3390/molecules31101586/s1. Figure S1. Panax vietnamensis var. fuscidiscus whole plant; Figure S2. Panax vietnamensis var. fuscidiscus (PVF) fresh root (**A**) and Black PVF (**B**).
