# Supplementary material for: Nephroprotective Effect of Black Panax vietnamensis var. fuscidiscus Against Cisplatin Toxicity
Source: Molecules. 2026 May 9;31(10):1586. doi: 10.3390/molecules31101586 (PMC13209401; doi:10.3390/molecules31101586)
Supplement: Supplementary file 1 [file molecules-31-01586-s001.zip › molecules-4262794-supplementary.pdf]

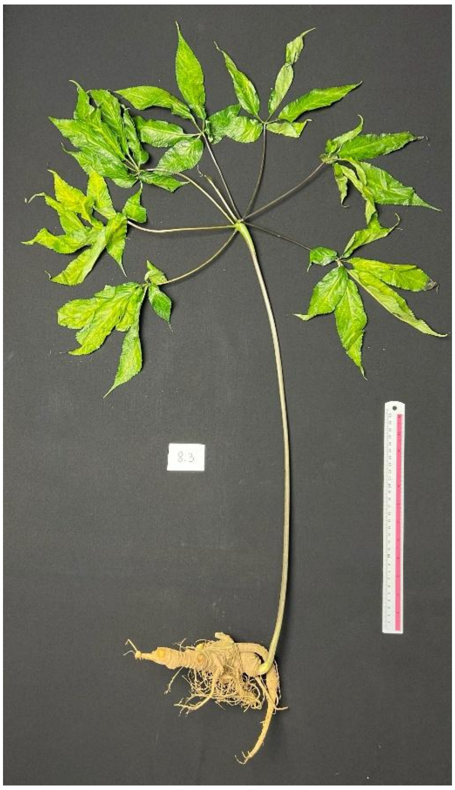

Figure S1. *Panax vietnamensis* var. *fuscidiscus* whole plant

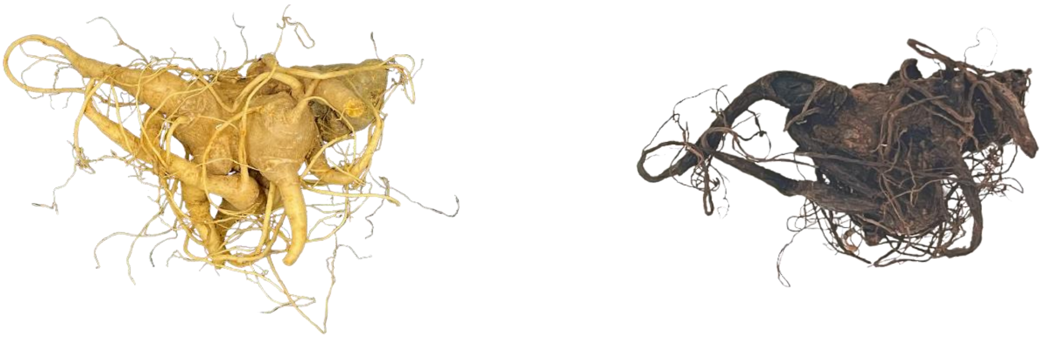

A B

Figure S2. *Panax vietnamensis* var. *fuscidiscus* (PVF) fresh root (A) and Black PVF (B)
